# Chronic Diseases in North-West Tanzania and Southern Uganda. Public Perceptions of Terminologies, Aetiologies, Symptoms and Preferred Management

**DOI:** 10.1371/journal.pone.0142194

**Published:** 2015-11-10

**Authors:** Soori Nnko, Dominic Bukenya, Bazil Balthazar Kavishe, Samuel Biraro, Robert Peck, Saidi Kapiga, Heiner Grosskurth, Janet Seeley

**Affiliations:** 1 National Institute for Medical Research, Isamilo Road, P.O. Box 1462, Mwanza, Tanzania; 2 MRC/UVRI Uganda Research Unit on AIDS, P.O. Box 49, Entebbe, Uganda; 3 London School of Hygiene and Tropical Medicine, London, United Kingdom; 4 Mwanza Intervention Trials Unit, P.O. Box 11936, Mwanza, Tanzania; 5 Weill Buganda School of Medicine, Mwanza, Tanzania; 6 Weill Cornell Medical College, New York, NY, United States of America; University of Oxford, KENYA

## Abstract

**Background:**

Research has shown that health system utilization is low for chronic diseases (CDs) other than HIV. We describe the knowledge and perceptions of CDs identified from rural and urban communities in north-west Tanzania and southern Uganda.

**Methods:**

Data were collected through a quantitative population survey, a quantitative health facility survey and focus group discussions (FGDs) and in-depth interviews (IDIs) in subgroups of population survey participants. The main focus of this paper is the findings from the FGDs and IDIs.

**Results:**

We conducted 24 FGDs, involving approximately 180 adult participants and IDIs with 116 participants (≥18 years). CDs studied included: asthma/chronic obstructive lung disease (COPD), diabetes, epilepsy, hypertension, cardiac failure and HIV- related disease. The understanding of most chronic conditions involved a combination of biomedical information, gleaned from health facility visits, local people who had suffered from a complaint or knew others who had and beliefs drawn from information shared in the community. The biomedical contribution shows some understanding of the aetiology of a condition and the management of that condition. However, local beliefs for certain conditions (such as epilepsy) suggest that biomedical treatment may be futile and therefore work counter to biomedical prescriptions for management.

**Conclusion:**

Current perceptions of selected CDs may represent a barrier that prevents people from adopting efficacious health and treatment seeking behaviours. Interventions to improve this situation must include efforts to improve the quality of existing health services, so that people can access relevant, reliable and trustworthy services.

## Background

The growing burden of chronic diseases (CDs), such as hypertension and diabetes, in low and middle-income countries, is an area of increasing concern [1–7]. This health transition is an additional strain on health systems already over-burdened with infectious disease management [5]. Efforts to understand people’s perceptions of these conditions have given rise to a large number of disease-specific studies aimed at informing health education and management approaches in Africa. This large body of work covers, for example, studies on the lay understanding of hypertension [8–13], stroke [14–21], diabetes [22–25] asthma [26–28] and epilepsy [29–39]. Some have suggested that lessons should be learned from the management of HIV in mobilising health services to manage the different CDs [40], particularly given the assertions that, due to the life-prolonging effect of anti-retroviral therapy, HIV should be considered a CD [41–43] and a condition that it is intimately linked to non-communicable diseases [44, 45]. While studies have shown that health system utilization is low for CDs other than HIV [46], it remains unclear how much of this usage of health facilities is related to differences in perceptions about these conditions or other factors, such as access to reliable and effective health services.

We conducted qualitative and quantitative research in southern Uganda and north-west Tanzania in order to collect background information for the future design of an intervention package aiming to improve services for the prevention and treatment CDs, including the formulation of educational messages to influence health seeking behaviour and service uptake. In this paper we describe the findings from that research on the knowledge and perceptions of CDs identified from rural and urban communities in the two countries. We collected data on the following conditions: hypertension, epilepsy, HIV, diabetes, cardiac failure, asthma and chronic obstructive pulmonary diseases. We did not investigate perceptions of and risk factors for other chronic conditions such as cancers, because the overall project had an a-priori focus on selected common non-communicable diseases. HIV infection was included due to its high prevalence, and its similarity with CDs with regards to service needs.

## Methods

Our complementary studies comprised: a quantitative population survey involving a random sample of community members, a quantitative health facility survey in the study communities, in-depth interviews (IDIs) in a subgroup of population survey participants and focus group discussions (FGDs) with men and women from the study areas. The purpose of this combination of studies was to triangulate findings from the different approaches; but also to generate additional knowledge beyond that which could be gathered using just one of these approaches. The objectives of the population survey were to determine CD prevalence, the prevalence of risk factors associated with CDs and to document self-reported treatment-seeking behaviour. An initial set of 12 FGDs was conducted just before the population survey commenced to establish the local terminology for CDs in order to inform the population survey questionnaire design and to develop the topic guide for the IDIs. A second more extensive set of 12 FGDs was conducted after the population survey to develop deeper insights into the thinking of members of the population on CDs and their views on the most appropriate resource for treatment. The objective of the IDIs was to explore such beliefs at the individual level, taking any personal or family disease experience into account. These interviews were conducted two to four months after the population survey was completed in each community where the interviews were to take place, Finally, the objectives of the facility study were to determine service readiness and utilisation of health facilities for these CDs and thus to possibly verify shortcomings reported by the public.

The methodologies of the population survey and the health facility study have been described elsewhere [46–48]. Briefly, for the population survey, a total of 2011 adults (≥18 years) were enrolled in Uganda and Tanzania, using a stratified, multistage sampling design with five strata in each country (one municipality, two district towns, two rural areas). Consenting adults were interviewed using an adapted version of the WHO STEPS questionnaire (WHO 2008) about their disease history and treatment seeking behaviours with regards to selected non communicable diseases and HIV infection, after which they were physically examined to determine the prevalence of these conditions and of CD risk factors.

For the health facility survey, a quantitative cross-sectional study of a representative sample of public and not-for-profit health facilities in urban and rural Uganda and Tanzania, including a total of eight hospitals, and 20 middle and 24 small sized health facilities, was carried out using an adapted version of the WHO SARA instrument (WHO 2013). We conducted structured interviews with facility managers, inspected resources and administered self-completed questionnaires to health workers. Study outcomes related to service readiness and provision regarding selected chronic diseases. Health facilities were located within the same geographical strata in which the population survey and the qualitative studies were conducted.

For the initial set of focus group discussions, we invited six groups of men and six groups of women from urban and rural settings, outside the main study area, in both Uganda and Tanzania. To obtain participants for FGDs the Tanzania social science team liaised with street leaders which are the lowest administrative authority in Tanzania and comprise individuals with usually profound knowledge of the neighbourhood. The street leaders were asked to identify people with the following key attributes namely: residents of study area, people who were knowledgeable about CDs based on their personal experience with CDs, including those who had suffered or cared for people with CDs, or who knew people with CDs. Similarly, the initial set of FGD participant names in Uganda was obtained through local council leaders, who again represent the lowest administrative unit. The Ugandan social science team asked the local council leader to identify the people who were residents in their areas. All the participants for the FGDs were to be persons aged 18 years or above. For IDIs, 116 participants were selected through simple random sampling from the population survey participants, and this sample included 46 individuals with and 70 without CDs. Few of the affected participants had more than one condition. The random sampling aimed to ensure the different study areas and the CDs under investigation were represented in the selection. Five epileptic patients (two from Uganda and three from Tanzania) were purposively sampled for inclusion after the random sample failed to include any epileptic cases. In order to limit interviewer bias, the interviewers were blinded about the health status of the sample composition and of individual participants.

For the second set of focus group discussions, participants were also selected from the population survey participants, maintaining the stratification of the population survey into rural areas, district towns and the municipality. Twelve groups divided on the basis of sex were convened in each country. Participants already selected for in-depth interviews were excluded.

The IDIs and FGDs were conducted using standardised topic guides. For Uganda, these guides were translated into Luganda while for Tanzania they were translated into Kiswahili. These are the languages spoken and understood by the majority of the people in the study areas.

Contact information of eligible IDI participants was obtained from the database established by the population survey. Community leaders together with population survey interviewers acting as tracers helped social scientists to identify the physical address (streets and households) of selected participants. They were contacted, and appointments for IDIs or FGDs given to consenting participants.

**Fig 1:**
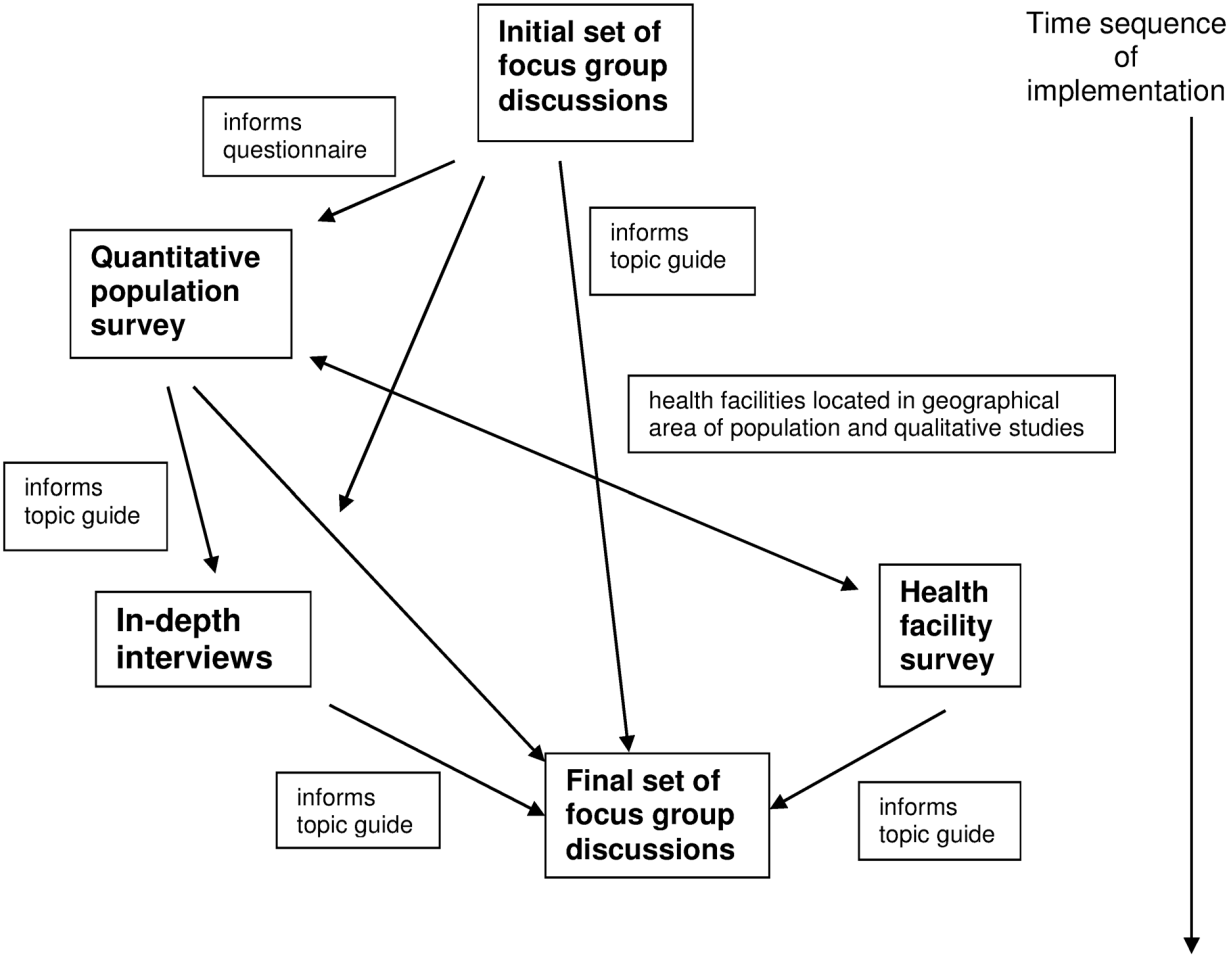
Flowchart of interconnected qualitative and quantitative studies.

To facilitate coding and analysis of data, all audio files with IDIs and FGDs were transcribed and translated from Kiswahili or Luganda to English. The translated texts were thematically coded using NVivo 9 Software (QSR International, Doncaster Victoria, Australia) to manage the coding. The coded thematic segments were compared and clustered to produce patterns and determine consistency or contradiction of responses. To complement qualitative data we used the population survey data set to extract variables on knowledge, perceptions, attitudes and treatment seeking for common CDs to facilitate coding. Fig 1, below, illustrates the way in which the different parts of the study data collection fitted together.

This paper focuses on the results from the qualitative studies. Results from the population and health facility surveys are reported in this paper when they confirmed, amended or contradicted qualitative results about public perceptions on CDs. The overall results of these surveys were published elsewhere [46–48].

### Ethical considerations

This study was approved by the ethics committees of the Tanzania National Institute for Medical Research, the Uganda Virus Research Institute, the Uganda National Council for Science and Technology and the London School of Hygiene and Tropical Medicine. Prior to interviews, study objectives and procedures were explained to all IDI and FGD participants and written informed consent was obtained (witnessed for non-literate participants). Participants were also asked if they agreed to IDIs and FGDs being audio-recorded. Where this permission was refused the interviews were written up from the interviewer’s notes. To ensure confidentiality of data, participants’ personal identities were not included in the transcripts or the analysis. In each country, data collection were conducted by trained research assistants, most of whom had attained a first degree in a social science discipline.

## Results

### Profile of participants of the qualitative studies

We conducted IDIs with 116 participants (≥18 years). Sixty of the IDI participants were from Tanzania and 56 from Uganda. Sixty-two of the IDI participants were women and 54 were men and there was a total of 46 participants who were affected by one of the CDs of interest for this research. Twenty nine of the affected participants were female and the remaining 17 were male. Among the 46 participants affected by CDs, close to half (22/46) were hypertensive, nineteen (19/46) had HIV infection, five were epileptic and two had COPD or asthma. There were two cases with more than one CD diagnosis. One of them had HIV infection and hypertension while the other had HIV infection and heart failure. This explains why the total number reported here is 48 rather than 46.

Forty of the IDI participants were recruited from rural areas, 38 from district towns and 38 from municipal areas. For the purposes of the analysis presented in this paper, participants from municipal quarters and from `district towns’ were combined as `urban’ into one group, as we found no evidence that their responses differed substantially.

Approximately 180 adults, both men and women, participated in the 24 FGDs. The participants were recruited from both rural and urban areas in both countries.

### Perceptions about what constitutes chronic diseases (CDs)

The term chronic disease was perceived to encompass broad categories of illnesses that either differed substantially from accepted biomedical definitions or combined elements of biomedical and local beliefs. For example, some participants from Tanzania described chronic diseases as ‘any disease which is incurable’, ‘any disease which does not easily respond to medicines’ or ‘any disease which afflicts many people’. One man said:

*CDs are those diseases which occur frequently in our community*, *those which are totally incurable*, *and those [diseases] which can only be temporarily relieved by drugs*.

(FGD male urban area Tanzania)

In Uganda, participants in one of the discussion groups expressed the view that a CD was a disease which remained symptomatic even after treatment.

In both countries, the term CD was often described to include recurrent, severe or endemic diseases. Thus when people were asked to list CDs, some included acute infectious diseases such as malaria, amoebic dysentery, tuberculosis and typhoid while others mentioned infections such as hook worm, bilharzia and sexually transmitted diseases, such as syphilis and gonorrhoea. Chronic non-communicable diseases such as asthma, epilepsy, diabetes, hypertension and other cardiovascular diseases were only mentioned after interviewers probed for additional examples.

### Perceptions of the magnitude and frequency of occurrences of CDs

When we asked about specific conditions, participants from both countries believed that there was a dramatic increase in the number of people suffering from hypertension, diabetes and HIV infection. The majority of study participants reported that they had seen patients with one of these three CDs. However, compared to Ugandan participants, relatively few participants from Tanzania reported that they had personally taken care of relatives or family members who had suffered from one of these conditions. In Uganda, participants from all of the second set of FGD sessions (6/6) and the majority of IDI participants (52/56) reported that they had cared for a relative with HIV-related diseases, hypertension or diabetes.

Participants who reported that they had cared for CD patients sought care from biomedical, traditional and spiritual healers or a combination of either two or all of these. Choice of care and treatments was influenced by multiple factors including, personal perceptions and beliefs about aetiologies, distance (both social and spatial) between patients and health care service providers. Health systems-related factors such as experience with frequent drug stock out coupled with a financial inability to buy missing drugs from private pharmacies/drug shop also encouraged CD patients to seek care from spiritual and traditional healers. Likewise, social marketing of health care products and services including promotion through electronic media of alternative therapies and spiritual healing influenced decisions on treatment choices.

Most of the participants from IDIs and FGDs who reported that they were aware of their CD status were those suffering from `hypertension’. This finding reflects data collected during the population survey, which indicated that hypertension was by far the most prevalent CD in both countries, with rates of about 23% in Uganda and 17% in Tanzania within the study populations[47, 48]. However, of 46 IDI participants, who had been diagnosed with a CD and had been notified about their CD status during the population survey (which had taken place about three months before the interview), 20 (43%) did not mention having received this information when interviewed during the IDI. This discrepancy was strongest amongst participants with stigmatized conditions such as HIV and those who had earlier reported a history of seizures or being on treatment for epilepsy.

### Local terminology used for different CDs

In both countries we found locally used terms to refer to different CDs. People used combinations of biomedical and local terminologies/concepts interchangeably to refer to a specific CD depending on the context. People living in rural and urban settings in Tanzania often used differing terminologies for the same condition, whereas in Uganda there were no marked differences. The majority of the terms were simply descriptive of symptoms or a metaphor for the condition (sometimes described by a phrase rather than a single word or term). In some cases biomedical terminologies were locally appropriated or adapted to refer to a CD. For example, hypertension was referred to as `pulesa’ (derived from ‘pulse’) in Uganda, and ‘BP’ in Tanzania. Table 1 summarises the terminologies and phrases used for different chronic conditions.

**Table 1:** Local terminology used in Tanzania and Uganda for chronic conditions.

Place	Language	Local term	Direct translation or meaning
**Asthma**			
Tanzania (urban)	Kiswahili	pumu	The formal Kiswahili term to refer to ‘asthma’
Tanzania (rural)	Kisukuma	machwi	Chest disease used to mean ‘asthma’
Uganda	Luganda	assima	Luganda word adopted from English term ‘asthma’
**Hypertension**			
Tanzania urban	Kiswahili	shinikizo la damu	Elevated blood, used to mean ‘hypertension’
Tanzania (urban and rural	Kiswahili	kuongezeka mapigo ya moyo	Elevated pulse/beats of the heart, used to mean ‘hypertension’
Tanzania (rural)	Kisukuma	chemba moyo	A term referring to the diaphragm
Tanzania (urban)	Kiswahili (slang)	bladi presha / presha	Kiswahili word adopted from English term ‘blood pressure’
Uganda	Luganda	pulesa	Luganda word adopted from English term ‘pulse’, meaning ‘hypertension’
Uganda	Luganda	obulwadde bw’omutima	A disease of the heart/a disease that affects the heart
Uganda	Luganda	Obulwadde bwe ntununsi	Disease causing palpitations
Uganda	Luganda	nutununsi	Shortened form of the above
**Diabetes**			
Tanzania	Kiswahili	kisukari	Kiswahili word adopted from English term ‘sugar’
Tanzania (rural)	Kisukuma	lutundagila	term that refers to frequent passing of urine (urination)
Uganda	Luganda	sukaali	Luganda word adopted from English term ‘sugar’
**Epilepsy**			
Tanzania	Kiswahili	kifafa	Convulsions
Tanzania	Kiswahili	degedege	A term to refer to convulsions (especially in childhood)
Tanzania (rural)	Kisukuma	lusalo lwa kugwa	Mental illness/madness; referring to epileptic fits
Tanzania (rural)	Kisukuma	lusalo lwa kupela	Mental illness/madness, referring to epileptic fits
Uganda	Luganda	ensimbu	Epilepsy
Uganda	Luganda	obulwadde bw’ebiggwo	Disease that makes people fall down
Uganda	Luganda	bw’omwezi omuggwi	Disease that occurs at the time of full moon (the time of falling)
Uganda	Luganda	obulwadde bw’omwezi ensimbu	Epilepsy that occurs at the time of full moon
Uganda	Luganda	obw’omutwe	Mental illness/madness, referring to epileptic fits
**HIV and AIDS**			
Tanzania	Kiswahili	ukimwi	AIDS
Tanzania	Kiswahili	virusi vya ukimwi	AIDS virus
Tanzania	Kiswahili	waathirika wa ukimwi	AIDS victim
Tanzania	Kiswahili	kale ka mdudu	Bug or insect (metaphor for HIV)
Tanzania	Kiswahili	kale ka ugonjwa ketu ka kisasa	A modern disease (metaphor for AIDS)
Tanzania	English	Juliana	Name of a brand /type of cloth commonly smuggled between Uganda and Tanzania when the HIV epidemic began
Tanzania/Uganda	English	slim	Descriptive term for the slimming effect of AIDS
Uganda	Luganda	ekiwuka	Bug or insect (metaphor for HIV)
Uganda	Luganda	akawuka	A tiny bug or insect (metaphor for HIV)
Uganda	Luganda	boomu ya mukuba	hit by a bomb, meaning ‘s/he got infected’
Uganda	Luganda	kamala byonna, telutariza	A disease that takes everything/ a disease that does not discriminate
Uganda	Luganda	mukeneya	A disease that causes one to become slim
Uganda	Luganda	siliimu	Luganda appropriated from English term ‘slim’
Uganda	Luganda	ekilya atabala	A disease that infects those who are unfaithful or promiscuous
Uganda	Luganda	embwa	Dog

People often used metaphors and euphemisms to refer to certain CDs—seemingly to avoid mentioning a stigmatised condition directly. Some of the terminologies used to refer to CDs were idioms or popular local terms that were introduced at a time when there was no formal vocabulary or direct translation of a certain CD. For example, in Uganda HIV/AIDS was sometimes referred using a local term “*kayovu*” or “*kayola*”. These were the local names for a banana pest which led to the destruction of many banana plantations in the areas around Lake Victoria in the 1990s when HIV infection became widely noticed. HIV/AIDS was the condition for which more alternative terms were given than for other CDs.

### Perceptions about causes and symptoms of common CDs

#### General observations

Participants in both countries shared beliefs about the causes, symptoms and the preferred treatment of common CDs. People believed that common CDs have multiple causes whereby both local/traditional and biomedical explanations of disease causation were referred to, as also reflected in Table 1 (further detail can be found in S1, S2, S3 and S4 files).

There was also a common belief in both countries that CDs such as epilepsy or asthma can be inherited, can be transmitted from one person to another or can be acquired as a result of supernatural powers.

#### Hypertension

Several participants from both countries associated hypertension with dietary habits such as eating raw salt, fatty foods, and gaining an excessive amount of weight. The most commonly reported symptom for hypertension was a sensation of increased heart beats. Other symptoms that were mentioned in both countries included headache, anxiety, depression, and sweating, especially during the night. Few participants knew that hypertension can be asymptomatic for a long time.

Some participants in Tanzania thought that hypertension might generally result from poor nutrition. A few participants in both countries observed that a lack of physical exercise could lead to hypertension. Other factors mentioned in Uganda that might be responsible for causing hypertension were: blocked blood vessels, holes in the heart, old age, working in polluted environments or stress. Financial and social-related stress and anxiety were reported to affect both rich and poor people in both countries resulting in hypertension. There was also a belief that hypertension was linked to wealth and affluence. One participant in Tanzania said “*huu ni ugonjwa wa matajiri”* (“blood pressure is a disease for the rich people”) *[…] “ugonjwa huu unawapata watu wa mijini”* (“this disease afflicts people from urban settings”*)*. Hypertension and diabetes were considered to be closely-related. The population survey data provided some additional insights into beliefs about symptoms and complications of CDs. Some Ugandan participants reported that swollen feet, weight gain, blurred vision, sharp chest pain, being easily tired and dizziness could be symptoms of hypertension. There were no significant differences between men and women in the reporting of these symptoms.

The population survey indicated that 17% (182/1095) of survey participants from Tanzania and 37% (343/916) from Uganda knew that hypertension can potentially lead to paralysis of muscles of the face or limbs (indicating a stroke). This knowledge was generally higher in urban than in rural areas (e.g. 60% in Uganda) [48]. However, participants in the IDIs and FGDs who had been diagnosed with hypertension from both countries, seemed unaware that a complication such as a stroke might be the first or only symptom of hypertension. One woman from Tanzania, who had reported being diagnosed first with hypertension and later suffered a stroke a few months prior to this study, said:

*I asked my physician*, *to explain to me the symptoms of hypertension; and the doctor told me “[…] hypertensive person may feel tired*, *sometimes she becomes weak*, *and she can also sweat excessively*.*” I am surprised because when I got a stroke*, *I was told that it was caused by hypertension but I did not experience any of these (other) symptoms* […]

(IDI female urban area Tanzania)

Some participants considered hypertension to be combination of diseases, as illustrated by an example from Tanzania:

*Blood pressure is [also called] heart disease[…]also known as asthma that is also blood pressure […] Yes*, *isn’t asthma also the same with heart disease*, *or the blood pressure*?

(IDI male, urban area Tanzania)

#### Asthma and other lung problems

Asthma was predominantly believed to be acquired through inheritance or through breastfeeding. For example, a participant from rural Tanzania said:

*Asthma is caused by some bugs*. *[…] if a woman breast feeds a child while she has this disease it is obvious that her child will acquire it*.

(FGD female rural area Tanzania)

While many participants were familiar with the term `asthma’, most confused it with other chronic lung diseases. One woman commented:

*I do not know whether asthma is the same as tuberculosis*. *I am a bit unsure*. *[…] They say [asthma] is like tuberculosis*. *Isn’t it*? *[…] The way I know if you have problems with breathing*, *I think that would be asthma*. *[*…*] You get instant constriction on the chest muscles*. *[…] you cannot breathe properly*, *or it can even reach a point where you become very weak*. *[…] it may also cause coughing; a person could cough pus [sputum]*, *[…]*. *We are used to calling it TB in Kiswahili but that is what others call asthma*. *So we do not differentiate among TB*, *asthma and tuberculosis (kifua kikuu)*.

(IDI female urban area Tanzania)

A woman participant from Uganda expressed a similar view

*I don’t know whether lung infection is a distinct disease*. *I don’t know whether tuberculosis falls under here or when someone has lung infection they have TB as well*. *I don’t know*.

(IDI female urban area Uganda).

In both countries none of the participants mentioned the allergic nature of asthma. However, in Uganda participants stated that cigarette smoking and working in a polluted environment could cause asthma. In Tanzania only a few study participants associated asthma with air pollution. Usually, they said, asthma can be caused by exposure to cold weather:

*[…] Asthma can be caused through exposure to the cold weather in childhood*. *[…] If a child is not well covered with heavy clothes*, *s/he will get cold*, *and the cold stays intact inside the body until later at adulthood when the cold becomes chronic and transformed itself into asthma*.

(FGD female urban area Tanzania)

This view was also expressed by some Ugandan participants:

‘*During cold weather asthmatic people do get difficulty in breathing and you can see them struggling to breathe*. *During that time*, *they breathe like chicks with flu”*,

IDI male rural area Uganda.

The most frequently mentioned symptoms of asthma were: body fatigue, difficulty breathing, elevated heart beat and general body weakness.

#### Diabetes

There was an overlap between the perceptions of the causes of hypertension and diabetes. In both countries, the causes of diabetes were, as with hypertension, attributed to individual lifestyles and particular nutritional practices such as an excessive consumption of fatty food. The majority of participants from Uganda believed that diabetes was specifically caused by eating too much sugary food while participants from Tanzania thought that it was both the excessive consumption of sugary and salty foods which could cause diabetes. A few people in Tanzania mentioned other causes of diabetes such as witchcraft or environmental pollution.

*[…] It is easy to believe that people with diabetes have been bewitched*. *[…] Hospitals cannot diagnose diabetes easily*. *[…] You can provide them with stool*, *urine or blood [samples] for examination but they do not see it […] until further investigation is done*. *During the period before the result is given*, *people would tend to think a patient has been bewitched*.

(IDI male, non diabetic, urban area Tanzania)

Most participants reported that they knew diabetic patients and thought that patients with diabetes frequently pass sugary urine (they based their belief that the urine was sweet on the observation that the urine attracted ants).

#### Epilepsy

Participants from both countries reported seizures to be the main symptom of epilepsy. The condition was believed to be associated with madness. Like HIV infection, epilepsy was a stigmatized condition. The stigma associated with epilepsy was reflected in the secrecy that surrounded the condition including the use of metaphors to refer to the condition (see Table 1 above):

*[…] Parents do not like to reveal that they have a child who has epilepsy*. *That is very rare to hear from the parents or relatives*. *[…] may be the relatives feel shy*, *probably because they associate it with sorcery*. *That’s why they feel shy*.

(IDI female urban area Tanzania)

Three among the five IDI participants who had been diagnosed with epilepsy during the population survey did not disclose that they suffered from epilepsy to the interviewers.

Several participants believed that epilepsy was contagious. A woman from rural Uganda said:

*I was told to use neither the cups nor the plates the epileptic person was using because I can contract epilepsy*. *We used to avoid this so much*. *She [a person in the family with an epileptic patient] would not let us use her utensils*.

(IDI female rural area Uganda)

Others in Uganda suggested that a person could acquire epilepsy by “smelling the fart of an epileptic person”, or getting into contact with the foam from the mouth of an epileptic person. Consequently, both family and community members were cautioned against helping someone during an epileptic fit for fear of contracting the condition.

In Tanzania, it was believed that epilepsy could be triggered by a worm (*minyoo*) swallowed during breastfeeding. A conversation with a participant who was said to have a relative with epilepsy presents this view:

*R*: *Epilepsy (degedege) normally is provoked by a worm that causes seizures (kifafa) during childhood […]*
*I*: *So*, *you mean degedege causes seizures (kifafa)*
*R*: *Yes*, *it is degedege which attacks a child’s brain during breastfeeding (at infancy)*. *It is a worm [and] if it is severe it can even cause a child to fall down*.
  (IDI female rural area Tanzania)

A few participants from Uganda believed that epilepsy can be inherited or was caused by witchcraft.

### Treatment seeking pathways for different CDs

Of the 2011 population survey participants (54% Tanzania; 56% Uganda; 54% men, 56% women), 150 (7.5%) reported that they had ever been told that they had hypertension or diabetes or that they had experienced seizures in the past. Of these, 19% of men and women in Tanzania and 8% of men and women in Uganda reported to have ever seen a traditional healer for these conditions. Differences between sexes were small; but percentages were lower in urban than rural areas in Tanzania and vice versa in Uganda.

In Tanzania, participants in the IDIs and FGDS said that hypertension and diabetes were mainly managed in district and regional hospitals. A preference for self-medication (through drugs purchased from pharmacies) was also reported, since public health facilities that were supposed to provide medication for hypertension were said to often run short of drugs, to have long waiting times and sometimes to be unfriendly places to visit because of negative attitudes of health workers.

*[…] For regular consultation I go to the [xxx] hospital*. *[…] it is a government hospital […]… but I also know one doctor who works there*, *so when I go there I do not get much trouble*. *[…] But to be frank*, *I cannot say the service is good*, *because if you do not know someone to assist you*, *you might stay there for hours without getting any treatment*. *[…] The shortage of drugs is not only found in dispensaries but even in that hospital*. *[…] they will give you two drugs and ask you to go and buy the rest from drug shops*.

(IDI male urban area Tanzania)

These reports were generally in line with observations made at various health centres during the health facility survey; and this was particularly the case at middle and small sized health facilities For example, of 24 facilities in Uganda and 20 facilities in Tanzania, only 9 (38%) and 5 (25%) respectively had medication for hypertension in stock, whilst this was the case for at least 75% of the four hospitals investigated in each country [46, 48].

Only 16 participants from the population survey in Tanzania and 38 from Uganda were currently on any medication for one of their chronic conditions. Of these, the majority (74%) reported that they were taking modern medicine only, whilst 13% reported taking traditional medication only and 13% said they were using both kinds of medications simultaneously. There were no substantial differences in these percentages between countries, but in Uganda more women than men reported the use of traditional medicines. There was no clear trend regarding differences between urban and rural areas. However, within these subgroups numbers were small and conclusions should be drawn cautiously.

Many IDI and FGD participants complained about the high cost of drugs for treating CDs. The health facility survey confirmed that patients would have to pay fees for treatment as part of the cost sharing policy in Tanzania or would have to buy drugs that were out-of-stock (in both countries). Data from the population survey indicated that informal payment demands from patients with CDs were, however, a rare event.

Apart from the utilization of modern medicine, treatment options for CDs mentioned in FGDs and IDIs in both Uganda and Tanzania included spiritual and traditional healing or self-medication with herbs, depending on the assumed causes of the disease. Unfortunately, no data are available on the use of spiritual healing from the population survey.

Choices were also influenced by participants’ confidence in the health care system. The use of modern drugs, for example, was reported to be influenced by the availability of drugs and the cost of accessing services (especially transport and the costs of tests, drugs and other user fees [in Tanzania]). There were some conditions and occasions when participants mentioned that people would not access treatment from health centres or hospitals, this was particularly the case for epilepsy. One participant from Tanzania said:

*People do not bother to take to the hospital a child who is attacked by epilepsy [degedege] […] because they know if you take a sick child to hospital*, *she will die [pause] what they do is to put her under the bed*, *make her inhale herbs*. *[…] People do say that in the hospital what they do is just provide a temporary relief*, *[…] We believe the herbs can cure it*.

[FGD, Female, urban Tanzania]

Other participants were inclined to seek faith healing for the treatment of epilepsy. One woman in Uganda said:

*I used it [traditional medicine to treat epilepsy] but it also failed*. *I now want to try prayers but I am afraid of getting attacks (by seizures) while attending the prayer*.

(IDI female rural area Uganda)

In both countries and for various other conditions IDI and FGD participants reported that at different stages of an illness patients would concurrently or serially employ traditional and biomedical approaches.

## Discussion

Our study illustrates how what people know and think about CDs can affect how they act. As observed in other studies, we found that the understanding of CDs drew on information and terminology gained from the biomedical sphere [8, 14]. Perceptions about causes, aetiologies and preferred treatment for common CDs were informed by multiple determinants. Traditional norms and community beliefs about the causes of CDs influenced individual choices of care and management of CDs. It is, however, apparent that although knowledge of treatment options is a necessary precursor for accessing care, other external influences including access to the services, and beliefs about treatment efficacy, influenced individual treatment seeking pathways. The understanding of most chronic conditions involved a combination of biomedical information, gleaned from clinic visits, and local informants and beliefs drawn from information shared in the community and the family [49]. However, our findings demonstrate that beyond the experience and beliefs of individuals, the societal and cultural context is important in shaping commonly shared beliefs and this understanding helps to explain the eclecticism which is practiced when treatment seeking choices are made [50–54].

The need to embrace the local understandings of disease causation in order to offer treatment options has been the subject of several other studies [9, 11, 25, 38, 53, 55–57], our findings show that this behaviour cuts across all the chronic conditions examined. While the sharing of new information about chronic conditions has yielded results in terms of the information that people now possess, that information should not obscure an appreciation for the existence of beliefs and tried and tested practices that may affect treatment seeking, and therefore influence the design and implementation of interventions. For example, health care providers need to be aware that in keeping with findings from studies elsewhere in Africa, we found that epilepsy was a stigmatised condition and, in addition, could not be treated by biomedical remedies. It was thought by some to be caused by witchcraft or evil spirits which affected treatment choices [39]. And, although HIV had been the target of many anti-stigma campaigns, our findings and the findings of other studies in East Africa show that it remains a stigmatised condition [58–60], which continues to influence treatment access and the sustained use of treatment.

There were several areas of similarity in the findings from the two countries regarding perceived causation, symptoms and preferred treatments for common CDs. In both places the terminologies used in clinical settings have been appropriated for use outside the clinic. It was therefore not surprising that biomedical terms were mimicked in local languages, referring to hypertension as ‘BP’ and ‘pulesa’ and diabetes as ‘kisukari’ and “sukaali’ respectively. Susan Reynolds-Whyte [57] documented similar naming practices in her research in Uganda and more broadly in Africa.

As already noted, in line with the context dependent eclecticism described above, awareness of CDs was not always accompanied by biomedical service utilisation. There was a wide usage of alternative care options both independently of biomedical care and in combination with biomedical care. Different approaches to care were influenced by many factors, as we have shown, including understandings of the cause of the condition as well as the availability of care at local health facilities. Indeed, other findings from the wider research project of which this study is a part show that most CD services, except for HIV infection, were concentrated at higher level health facilities, which were not always readily accessible to participants [46, 48, 61]. A recent study from Uganda which looked at health care utilisation for all conditions, carried out in one of the same districts as our study reported a high rate of the use of alternative care including the private purchase of drugs from pharmacies and drug shops and use of herbal remedies although all respondents were aware of the existence of local government health services [62]. Drug stock outs, costs of services and long distances to facilities, were mentioned as barriers to the access to health-facility based care, in keeping with our own findings.

The inequalities in access to health care because services are not available at local health centres and the services that are available are poor, has been highlighted by many recent commentators. For example, in their recent `viewpoint’ on the quality of care during the Ebola outbreak in West Africa, Boozary, Farmer and Jha [63] observe that `when people receive care that is unsafe or ineffective or they are not treated with respect, it is little surprise they avoid further care’ (p.E2). This structural violence of inequality in access to health care [64], inherent in health systems where reliable and trusted services are not available to the socially and economically marginalised [65], has a profound impact on health seeking behaviours; this rather than knowledge of disease aetiology greatly influences access to care. This contextual understanding of public health facilities and services, both in terms of access and quality of care, and the social and economic situation of those who may need care is essential in order to understand how people view care services and may utilise different sources of care [66–68]. Barriers to access may be compounded for non-communicable diseases, which can often be seen as being associated with modifiable life-style factors [69], if survival is viewed as a person’s life-style choice (to lose weight or stop drinking alcohol, for example). As Marsland and Prince [70] observe `access to choice depends to a large extent on the means people have at their disposal and the environments in which they live’ (p. 458). The underutilisation of health services for chronic conditions needs to be seen in this context. Knowledge is not enough; trusted and appropriate services need to be accessible and reliable.

A strength of our study was that we were able to compare information from qualitative work, conducted through FGDs and IDIs, with findings from quantitative investigations: epidemiological and health facility surveys in the same population and about the same time. This allowed us to triangulate and cross-check information across the different studies. Another strength is that all these studies were in two different East African countries, using standardised research protocols. Our observations were strikingly similar across the two populations. The importance of understanding how people not only view the causes of these conditions, but also hold perceptions of the efficacy of different treatment options can guide the design of interventions to improve health care for CDs in East Africa and elsewhere. A first step towards this is raising the awareness of, and sensitivity to, different understandings of causality of CDs of the health facility staff, so they make fewer assumptions about what their patients may believe about the treatment they are offered.

In summary, local beliefs and perceptions as well as importantly, the availability of services, for CDs represent a barrier for people in our study areas accessing health accessing care. Efforts to improve this situation should focus on the quality of existing health services for CDs in order to improve the quality of services as well as the trust among the population towards those services, while also taking account of community as well as patients’ beliefs about CDs.

## Supporting Information

S1 File Topic guides. (PDF)

S2 File Focus group discussions. (PDF)

S3 File IDI–rural analysis summary. (PDF)

S4 File IDI–urban analysis summary. (PDF)
