# Supplementary material for: Chronic Diseases in North-West Tanzania and Southern Uganda. Public Perceptions of Terminologies, Aetiologies, Symptoms and Preferred Management
Source: PLoS One. 2015 Nov 10;10(11):e0142194. doi: 10.1371/journal.pone.0142194 (PMC4640879; doi:10.1371/journal.pone.0142194)
Supplement: S1 File — (PDF) [file pone.0142194.s001.pdf]

S1-Topic guides

Interview Guide for in-depth interviews

1. Ask about participant's background, who they live with, what they do for a living. Probe for education level, marital status, religion and tribe.

2. Explore which signs and symptoms are associated with which illnesses – list as many different illnesses as the respondent knows, including the local names for the illness. Probe to see which symptoms are linked for different diseases. [Mention signs for each illness; one at a time]

3. What sort of treatment would the respondent suggest for each illness listed? Probe for western, herbal/traditional and spiritual treatment options. [probe for each illness]

4. Ask if the respondent has any long standing illness/health problem AND/OR personal experience in caring person with chronic disease; If YES, probe: what type of illness, how long has s/he suffered from the disease?; Probe if it limits activities of the patient in any way? (Probe for physical and social limitation); attitudes of family members & health care providers towards the patient.

5. Has the respondent any experience of seeking treatment for any of these illnesses her/himself or someone else (a relative), what can they say about that experience? Probe for: Type(s) of treatment sought and reasons for each choice? Probe for: accessibility to the services (e.g. distance to the facility, cost of treatment), frequency of visits to the facility, who decided when and where to go for the services?

Guide for focus groups

Each group discussion can focus on the symptoms below grouped according to different NCDs (if necessary) or restrict discussion to certain symptoms, resulting illness and treatment seeking.

Using the checklist below of different symptoms explore which are associated with which illnesses – list as many different illnesses as the group know, including the local names for the illness. Probe to see which symptoms are linked for different diseases.

- Head ache
- Disturbed vision/ Visual blurring
- Dizziness
- Seizures,
- Loss of consciousness,
- Confused speech,
- Paralysis of muscles of face, arm and leg
- Pain in the chest and/or left arm, collapse
- Increased thirst and fluid intake, not explained by physical efforts or warm environment
- Frequent need to urinate, also in the night
- Unexplained weight loss
- Weakness
- Genital eczema (thrush)
- Drowsiness,
- Vomiting
- Deep breathing
- Loss of sensitivity for touching and pain e.g. in parts of feet or hands, numbness, tingling, pain
- Loss of eye sight, unexplained blindness
- Ulcers of foot
- Deformed feet acquired during life without accident
- General fatigue
- feeling to be easily short of air when working
- need to sleep with the upper part of the body elevated
- cough at night with watery or pink frothy sputum
- wheeze
- increased breathing frequency
- swollen legs
- nausea
- lack of appetite
- swollen belly
- engorged pulsating neck veins
- bluish colour of lips
- usually history of asthmatic fits

- aura: happens before the seizure (strange feelings or sensations)
- confusion and drowsiness (after seizure)
- seizures resulting in injuries: wound of the tongue, head injury
- history of earlier seizures
- loss of body weight
- diarrhoea
- night sweats
- fatigue
- fever
- painful inflammation of the mouth, difficulty eating
