# Supplementary material for: Chronic Diseases in North-West Tanzania and Southern Uganda. Public Perceptions of Terminologies, Aetiologies, Symptoms and Preferred Management
Source: PLoS One. 2015 Nov 10;10(11):e0142194. doi: 10.1371/journal.pone.0142194 (PMC4640879; doi:10.1371/journal.pone.0142194)
Supplement: S2 File — (PDF) [file pone.0142194.s002.pdf]

S2- Focus Group discussions

Known CDs	Signs and symptoms	Causes	Treatment options	Prevention
Rural males				
Epilepsy	Seizures, a crying bird triggers the yabwe, reduction in brightness, bent lips, lameness of some body parts,	Genetic, high fever ''yabwe'' (convulsions), witchcraft, going near an epileptic victim, when s/he farts and you smell that you also contract epilepsy, getting in contact with the foams from an epileptic person	Traditional but only cure it the one originating from convulsions. When one fails in fire, it will never be cured,	
Diabetes	Weight gain, swelling of the feet/joints, if one is fat, loses weight, body weakness, frequent urination, limited sweating, pricking sensations,	Taking sweet things, taking sweet fruits like sugar cane, pawpaws, don't know, not taking sugar during old age you get diabetes, taking too much sugar as one ages.	Western, herbal although have no test kits. Prayers combination of western and prayers/spiritual	Avoid eating sweet things, not eating sweet fruits, for low sugar levels eat sweet fruits. Physical exercises
Asthma. Only affects children but not adults	Cough, difficulty in breathing, chest pain, suffocation,	Untreated cough, dust and then there after becomes genetical/hereditary	Western only gives relief but not cure, traditional if you get it cures it.	

HIV	Hair loss, weight loss, herpes-zoster, boils,	Unprotected sex, use of unsterilised equipment, mother to child transmission	Western, traditional but first go the hospital and get tested, spiritual	faithfulness, condom use,
Lung infection (COPD). An infection around the chest	coughing	Riding without protective gear. The lungs get holes due to the wind, cigarette smoking, dust, too much cold		
Hypertension. When ones gets blood pressure attack, people say one got a heart disease.	The becomes weak, getting tired quickly, can't go up hill easily, sweating a lot getting attacks when upset,	Weight gain, eating fats that narrow the blood vessels, worries, about debts, bad eating (eating beef for a lot duration),	Western, herbal, spiritual,	Avoiding being upset, eating fatty foods and worrying,
Heart failure related to hypertension				
TB and asthma related but could not tell the difference. TB is infectious while asthma is not	Productive cough, chest pain			
Rural females				
HIV	Painful legs/joints, herp-zoster, weight loss, boils, diarrhea, sores, natural skin loss, rashes, energy loss,	Unprotected sex, use of sterilized equipment, mother to child transmission,	Western, traditional but first test from western hospitals. Use of	condom use, faithfulness, HIV only thrives in

			traditional medicine follows thinking that one is bewitched. Use of traditional medicine happens before one gets to know what is suffering from	particular blood groups
Asthma linked to TB all attack the chest and cause coughing but TB has no cure	Suffocation in the morning and evennning, coughing	Genetical,	Traditional,	
Epilepsy	Seizures, foams, urinatiation during seizures,	Going behind an epileptic person during an epileptic attack, touching an epileptic person during an attack, witchcraft, fever that attacks the brain,	Western, herbal, spiritual, all the three,	
Hypertension/ Heart failure	Seizures, collapsing,	Worries, being upset,	Western, spiritual and medication	Nothing to do
COPD		Much alcohol, cigarette smoking,		
Diabetes	Frequent urination, athletes' feet, wound take long to heal	Taking sweet things,	Western, herbs,	Nothing

TB	Chest pain, cough			
Peri-urban males				
Pressure/ Heart failure. Related to each other same causes		Worries, so many thoughts, over working, eating a lot of fatty foods, having a lot of money when one buys a lot of fatty foods,	Western, rarely use traditional medication, prayers but very hard	Exercises, reducing fatty foods' intake, worrying less, reducing body weight,
Asthma/COPD/lung disease		Smoking (cigarettes, wind pipes), , coldness, riding on motor cycles without wearing proper gear, HO2 enters the lungs, taking alcohol, polluted environment,	Western, herbs, churches,	Proper dressing, sensitising people, reduce charcoal stove usage,
Diabetes	Genetic	Taking sweet fruits like mangoes, sugar canes, too much sugar, taking a lot of sugar as one is aging.	Western, herbs but take long to respond to treatment, spiritual because nothing God fails,	Taking a balanced diet, eating sour foods like red pepper, going for medical checkups,
HIV/AIDS Also still stigmatized.		Unprotected sex, unsterilized equipment, mother	Western, traditional but mainly before learning what	

		to child transmission,	one is suffering from. Spiritual (prayers and witchcraft) and all the above. Nothing fails God	
Epilepsy. Epileptic person working as a bodaboda .Stigmatized cos people don't want to come out and share. When an epileptic person falls in fire, he does not survive death	Comes like an evil spirits	Genetic, one is born with a ''ekiwuka'' in the head, helping an epileptic person during an attack before s/he regains consciousness. Genetic and bewitched belief, sharing meals with an epileptic person	Western presently but used to go to witch doctors, herbs and churches,	
Peri-urban females				
Pressure/heart failure	Seizures, dizziness, high palpitations, high heart rate, feet swelling, dizziness,	Cholesterol, stress, worries, thoughts, lack of exercises, being over shouted at, much dirt,	Western, herbal, spiritual and all the above	Medical checkups, avoiding fatty foods, increased green consumption,
HIV/AIDS	Sores, skin rash, herp-zoster, hair loss,	Unprotected sex with an infected person, accidents, use of unsterilized equipment, mother	Western, traditional and spiritual	

		to child transmission,		
Epilepsy	Seizures, frothing, body weakness,	Chicken pecking baby one into the head, Witchcraft, accidents, farting, from an epileptic person, HIV virus causes, epilepsy, fever like meningitis, helping epileptic individuals during an attack,	Western, traditional ,	
Asthma/lung infection/TB/pneumonia	Coughing, suffocation, fever, ,	Coldness exposure, hereditary, exposure to cold weather, alcohol taking, smoking	Western, local, spiritual,	
Diabetes	Self-injection, unhearing wounds, swelling feet, frequent urination, blurred vision, body weakness, dizziness,	Too sweet things, sugar over taking, eating sweet potatoes, bananas, biscuits, cakes, sugary fruits eg mangoes, sugar cane, ‘’dirty heart’’ meaning none use of cleansing medication beyond six months, over eating	Western, tradition and spiritual and both	Not eating sweet potatoes, cassava, banana, reduction on the quantity of the food consumed

Urban females				
Diabetes same as pressure,	Frequent urination, body aches, paralysis, stomach ache, feels thirsty all the time, joint aches, wounds take long to heal, body swellings,	Taking a lot of sugar, fat foods, eating raw salt,	Western, herbal, sprayers, collaboration between herbal and western,	Eating a lot of greens,
HIV	Red lips, hair loss, weight loss, diarrhea, loss of appetite, persistent fever sores, body rashes, cough,	Tooth extraction, unprotected sex, use of unsterilized equipment,	Western treatment, herbal,	Condom use, HIV testing, reducing sexual partners,
Pressure/heart failure	Paralysis, headache, hates being frightened, body swellings, over sweating, loss of senses, swollen body parts, general body weakness,	Worries, a lot of fats occupying the heart, blocked blood vessels, over eating fried foods,	Western, herbal,	Not eating fried foods, eating raw bellies,
Asthma/TB/lung infection/pneumonia/COPD	Difficulty in breathing, cough, sneezing a lot,	Skull diseases, smoking, hereditary,	Western,	
Epilepsy. Does not get cured when he falls in fire.	Seizures, foaming, dizziness, startle,	Can contract it when you smell an epileptic person' fart, Skip over his urine u also contract epilepsy, hereditary, fever leading to convulsions, affecting the brain	Western, local and spiritual	While n treatment, the epileptic patient is kept out of reach of people because when you are close to him, you also

				contract the diseases
