# Supplementary material for: Chronic Diseases in North-West Tanzania and Southern Uganda. Public Perceptions of Terminologies, Aetiologies, Symptoms and Preferred Management
Source: PLoS One. 2015 Nov 10;10(11):e0142194. doi: 10.1371/journal.pone.0142194 (PMC4640879; doi:10.1371/journal.pone.0142194)
Supplement: S3 File — (PDF) [file pone.0142194.s003.pdf]

S3- IDI – rural analysis summary

CD	Causes	Signs and symptoms	Treatment type
Asthma	If one was not covered very well while he/she was young	wheezing	Herbalists
Asthma	smoking cigarettes and sharing cups	difficulties in breathing	herbal
Asthma	very bad chest infection that is not well treated	cough all the time, even when the cold comes they suffocate	
Asthma		Eyes become very white and glittery<br><br>when it is rainy, they have to cover themselves	traditionalists boil herbs,<br><br>medical treatment
Asthma		get difficulties in breathing	Garlic, Medical treatment
Asthma	it is hereditary	Falling down<br><br>difficulty in breathing	Medical, herbal but does not heal completely
Asthma	It's genetic	Hardship in breathing	Medical treatment
asthma	It's contagious (eating and drinking with and infected person)	breathing faster, his eyes turn red, and he loses peace of body, shivering, and other cough	Western, prayers doesn't help same applies to herbal medicine
Asthma		During cold weather you get suffocated in that you cannot breathe very well, whizzing on	Go to hospital, herbalists, others are prayed for and get healed.

		your throat like the way a cat does	
Asthma	In born	suffocate when it is cold,	Majority use herbs and very few go to hospitals.
asthma		gets difficulties in breathing during poor conditions like cold weather	traditional medicine,<br><br>western hospitals for treatment
Asthma		person gets difficulties in breathing during the cold conditions	Herbalists , prayers, western
asthma		patients normally are to sensitive to coldness, the patient will struggle to grasp breath, they become restless	
Asthma/TB		whizzing sound from the chest, coughs	Western medicine
Diabetes	drinking too much sugar, eating sweet things like sugar cane,	Fat, unable to run, have no strength	Traditional healer claim to heal t but they don’t,<br><br>prayers
diabetes	abnormally swollen	Putting on weight or getting too fat things like that	boil flowers and drink, raw sugar mixed in water and you give him to drink up

diabetes	taking too much sugar		
diabetes	eating too much fat	Some people swell feet	daily injections, tablets, Modern medicine
diabetes	May be taking a lot of sugar or may be taking sweet beverages		Medical treatment,<br><br>No traditional medicine.
diabetes	taking sugar	feet swell, get pain in the feet as if they are stepping on thorns	Medical treatment, herbal, others go for prayers but not sure if the heal.
Diabetes	using so much things a lot of salt or sugar	a skin irritation every time,<br><br>swollen legs, joint pains	Medical treatment
diabetes	taking a lot of sugar, or eating very sweet foods		Medical treatment, Some people go for prayers but don't get cured
diabetes	eating sweet foods, taking too much sugar		
Diabetes		Sweating	western
Diabetes	Too much sugar or little sugar in the body.	the stomach swells, and diabetes leads a person to lose weight and another thing the hair can all get pale.	Western , traditional herbs

diabetes		his legs swell, his fingers swell, and his heart beats faster all the time, sweat a lot	
diabetes	it is inherited	most of the times they swell the feet, they sometimes also get sores in the soles of their feet and usually get fat	Western, some go traditional healers but don't improve
diabetes	eating sweet foods, taking a lot of sugar,<br><br>taking excessive salt		
diabetes			
diabetes	drinking too much sugar and alcohol, also eating raw salt, eating a lot of salt	a person is sick all the time and injects himself daily, forbidden not to eat thing like salt, sugar and drinks a lot of water	Medical treatment,<br><br>Traditional medicine but so sure about it., prayers at times patients are prayed for and get cured.
epilepsy		falling down suddenly and produces saliva, discharging gasses	
epilepsy	witchcraft, and others say it is inborn in a family	One falls down; they pass out gas and urinate.	when a person dies of epilepsy all their belongings are put on their

			grave saying that someone might be infected with it also<br><br>Unless one has just got it that's when they can get cured but when they fall in fire and urinate on themselves it is said that one does not get cured. Western medicine.
epilepsy	Mental issues when things are not going right.	Falling down	Western no traditional medicine that works
epilepsy	inherited in familie**s**	falls, they foam, and when they are recovering from the attack, they soil themselves	prayers
epilepsy		falling down, produce saliva, urinates on himself .	Medical treatment
epilepsy		feels dizzy all the time, they get an attack and fall down when a new moon comes,	

		other than falling down, convulsing, frothing while others vomit	
epilepsy	It's hereditary	falls down and froths and they convulse,<br><br>the day she will get an attack, she is very weak	medication from the health center
epilepsy		you he falls down, he produces foam in the mouth, looks unconscious	some people go to herbalists and get relieved<br><br>Medical treatment helps especially in early stages.
epilepsy	evil spirits.	fall down, they froth, kicking around and get convulsions	Western but majority go to traditional healing.
epilepsy	Poor blood flow in the body	fallen there and the eyes pop out and convulse	modern medicine,<br><br>traditional healers<br><br>prayers
epilepsy		falls down, he urinates on himself or emits saliva	Western and herbalists
epilepsy	hereditary disease	they fall and get stiff, saliva comes out of the mouth, and sometimes the eyes balls open so wide	Herbalists,<br><br>Western treatment

epilepsy		makes some sort of noise noise and then goes down, foam coming from the mouth, loses consciousness	
epilepsy		falls down,<br><br>urinating on himself, foams in the mouth, and a lot of tears come out until he gains consciousness	
Epilepsy	if their saliva gets in contact with you, you also acquire it		
heart disease	Worries due to many problems	Shock and being scared	
heart disease	smoking cigarettes		
heart disease	result of the fatty things we eat, worrying too much	breathes so much, and their heart beat is faster	Prayers and western treatment
heart diseases		whole body weak, difficulty in breathing, having excessive body fluids, respiratory functioning abnormally	
heart failure			western

heart failure	Some veins of the heart being dead		traditional herbs,<br><br>western medicine
Heart failure		stunted growth	
heart failure	having a lot of thoughts		Medical treatment
heart failure	worried so much it makes the heart restless		Both western and traditional medicine. Some go for prayers.
heart failure		patient feels palpitations all the time	Western<br><br>Doesn't trust local medicine
Heart problem	When someone does not drink a lot, when there insufficient water in the body**.**		Eating sour eggplant (katunkuma), western medicine, eating green bitter vegetables
Heart problem		Palpitations of the heart are very high.	a person with heart problems is taken to India for an operation
HIV	sexual intercourse	Frequent sickness, his hair color changes, his skin pales	western
HIV	Sexual contact with an infected person	lose of weight, skin rash, red lips	

HIV		Constant fever, red lips, loss of weight , cough	Western medicine, traditional one doesn't work/ not effective
HIV	Unprotected Sexual intercourse, injections,	Frequent fever, cough, headache, diarrhoea	Medical treatment,<br><br>Prayers but doesn't confirm
HIV	sexual intercourse with an infected person	got sick, grew thin,<br><br>herpes zoster but today, it no longer attacks,<br><br>, you just see a HIV positive person growing thin and since people take HIV treatment, he/she dies without notice.	Medical, herbalist help to get relieved but not cure
HIV	Promiscuity, blood transfusion and unprotected sex.	Lose weight, they can get running stomach, getting a skin rash, for some, their hair loses its texture and color.	Medical treatment and are given ARV's, herbal medication
HIV	having a sexual relation with a person who is infected without first knowing each other's status	skin rash, persistent cough, persistent fevers, the skin becomes pale, skin tend to change and their lips change to pink, complain of wounds in the mouth all the time	Medical treatment ,<br><br>herbal (kadomola),<br><br>prayers
HIV	Extramarital sex and using unsterilized syringes	persistent fever for over a months, becomes weak, some people are noticed through their mouth, their lips crack, their	Western , prayers relieve you of your thoughts,

		bodies pale, they develop skin rashes and other are attacked by herpes zoster	
HIV	Having a sexual relation with any other person, Sharing sharp instruments like needles, safety pins and razor blades.	constant fevers, frequent cough, getting a skin rash including diarrhoea herpes zoster, reddish lips, decolorizing of the hair and eventually hair getting off.	Medical treatment though some few go for herbal treatment and traditional healing.
HIV		loses too much weight, they have skin rash, the lips become red, and the hair gets brown and some also get their stomachs swelling	traditional healers, medical treatment, miracle healing in the saved churches
HIV	sexual intercourse without using a condom,<br>razorblades and knives	herpes zoster, loss of self-worth	You go for medical treatment, local/traditional medicine
HIV	Sexual intercourse between an infected and an uninfected person.<br>Shared things like injections, razor blades and other sharp things.	get herpes zoster, being bedridden,<br>suffering from many sickness at ago like flu, headache, chest pain/cough	

HIV	sexual intercourse with a person without using a condom,<br><br>if you have a wound or you get involved in an accident and your blood comes into contact, through other instruments like razor blades and safety pins and through the machines used in the hospitals to extract teeth	he may suffer from dry cough that does not heal, he may be attacked by herpes zoster, he may have diarrhoea and persistent fever	Medical treatment, other healers can't treat it instead worsening the condition
HIV		blood pressure comes down and become bed ridden and can't even come out of bed	Traditional medicine that are trying but it's not effective, prayers
HIV	Having sex without a condom with an infected person or sharing skin piercing instruments with an infected person such as a razor blades that can cause you get infected.	Sores, boils, someone losing too much weight, become pale, some lose hair and it gets worn out.	Western, prayers and traditional healers do not work to treat HIV.
Lung			Western
lung diseases	smoking cigarettes, smoking marijuana	Cheeks, arms and legs swell.	Western,
lung diseases	smoking the pipe, drinking alcohol	Difficulties in breathing	Western, some go to traditional herbalists but don't get cured

Lung infection	born with weak lungs, smoking cigarettes	Un confortable in breathing, coughing,	Medical treatment
Lung infection	smoking cigarettes and taking alcohol	chest pain, pain inside and his heart would be restless ns,	
lung infections	one takes too much cigarettes, his lungs get holes and also taking a lot of hard alcohol (walagi kasese and cigarettes		
lung infections	Smoking cigarettes	feel pain in the stomach and you discharge a bad smell	Medical treatment
lungs disease			
Pressure	Much thought,	Frightened, recurrent sickness, general weakness	Western traditional but don't believe it works, prayers
pressure	Being Worried	Falling down,<br><br>signs of diabetes are similar with those for pressure	go to hospital to be given tablets, and also reduce worrying,<br><br>do not eat salt,<br><br>traditional medicine Some say it is effective, others say it is not, prayers reduces it slowly , traditional medicine but western medicine works better

Pressure	eating very fatty foods	Get startled and fall down	
Pressure	Thoughts or worries	heavy breathing, sweating	not to mistreat a person with pressure<br><br>Traditional herbs that are boiled
pressure	Don't know	Sweating,	
pressure	getting worried, eating fried foods	Hears things making noise, it can make them feel bad, they get scared and fall down.	Medical treatment and herbal
pressure	Thinking too much, being worried	get a pain in the heart and they faint, fall down and faint, high palpitation of the heart	Hospital for treatment, herbal medication
pressure	being fat, eating fatty foods Like meat, taking undiluted milk, drinking beers, eating fried foods every, eating raw salt.	Sweating ,<br><br>feel headache, feel aches in the ribs	western hospitals, traditional medicine to get relief. Some go for prayers but don't work for them.
pressure		very many thoughts, getting worried, become very fat and you are unable to walk, eating fatty foods	Both western and traditional especially those poor ones who can't afford to buy drugs from the hospital.
pressure	Eating fatty thing all the time, worries		modern medicine, traditional herbs

Pressure	Taking too much oil (fatty things), and having a lot of thoughts	palpitations and sweat all the time	western hospitals,<br><br>local medicine
pressure	when one has a lot of fat in their bodies	get paralyzed in some parts of their body	Getting away from noisy place to calm ones, not to eat fatty things , western treatment, traditional practitioners
pressure	stress like for example if you secured a loan from a bank,<br><br>getting frightened,<br><br>losing a very helpful person like a child or a parent,<br><br>lot of fats in the body	heart beats so fast, when he climbs a steep slope,<br><br>Any abrupt occurrence he gets frightened	Western, With prayers you get relieved of stress and when the stress reduces, pressure is also likely to heal,<br><br>Take lemon to help to reduce the fats, reducing on the consumption of fatty foods.
pressure		Sweating, BP level is higher	
pressure		a person sweats, very fat but these days even small ones get the blood pressure	
