# Supplementary material for: Chronic Diseases in North-West Tanzania and Southern Uganda. Public Perceptions of Terminologies, Aetiologies, Symptoms and Preferred Management
Source: PLoS One. 2015 Nov 10;10(11):e0142194. doi: 10.1371/journal.pone.0142194 (PMC4640879; doi:10.1371/journal.pone.0142194)
Supplement: S4 File — (PDF) [file pone.0142194.s004.pdf]

S4-IDI urban analysis summary

CD	causes	Signs and symptoms	Treatment type
Asthma	Smoking		
Asthma		Struggles to breath	Western e.g. inhalers and local garlic and honey
Asthma	Flu and cold conditions	Difficulty in breathing during cold conditions	
Asthma		Difficulty in breathing,	Western. Long diagnosis procedure discourage patients from continued treatment seeking
Asthma	Cold weather	Difficulty in breathing, seizures, snoring	Western (injections, inhalers)
Asthma	Genetic, sharing utensils, inhaling dirt		Western and local
Asthma	Cat fur in the food		Western and herbs
Asthma		Cough, feeling cold all the time	Western
Asthma	Walking in the cold, environments		
Asthma, TB COPD: all affect the lungs	Cigarettes	Slim, breathes like a cat, cough, heavy breathing	Herbs, western and prayers
Asthma, TB, Pneumonia, COPD	Smoking	Short breathing intervals, cold weather intensifies it, cough	Western and herbs
Asthma/COPD	Cold weather, holes in lungs	Difficulty in breathing	Traditional
Asthma/lung infection	Weather conditions	Difficulty in breathing during cold weather, persistent cough,	Herbalists claim to cure it. Western because they are diagnosed from there
Asthma/lung infection (respiratory)	Heredity, being near fumes/charcoal, smoking cigarettes,	Difficulty in breathing because of Blocked nose, cold weather, chest pain,	
Asthma/lung infections	Smoking cigarettes	Difficulty in breathing	

COPD	Smoking	Blocked throat, chest moves up and down, air shortage	Traditional and western
COPD	Smoking, alcohol, polluted environment		
COPD		Flu	Western
COPD	Cigarette smoking, alcohol and environmental pollution		
COPD	Smoking and alcohol taking	Hard breathing	
COPD	Smoking, drug abuse		
Diabetes			
Diabetes	A lot of sugar	Paralysis, over sweating, burning fire sensation	Western medicine, herbs, crude waragi
Diabetes	A lot of sugar	Weight gain/loss, itchy feet	Western and local
Diabetes	Sweet foods	Paralysis	western
Diabetes			
Diabetes	Taking a lot of sugar, alcohol,	Self injection, don't take sugar, paralysis of the legs, bad vision	Western,
Diabetes	Diets, a lot of sugar,	Frequent urination,	Western, herbs cure (hear that over the radio), mostly go to born again churches. Pray but it does not get cured.
Diabetes	Taking sugar,	Taking less sugar,	Western, but because they get the same drugs every time they go the hospital and have been on medication for long, they decide to go for herbs
Diabetes		Blurred vision, frequent urination, energy loss	Herbs have no side effects and are nutritious
Diabetes	Taking too much sugar	Feet swelling,	Western, spiritual,
Diabetes		Sweating tiredness, cough, weight loss and gain	

Diabetes		Stroke, decolouring lips, skin darkening, dry skin, swollen feet, blisters which later turn into wounds, weight loss	Western and herbs but have to go there early before it becomes dangerous majority herbs, western, has many side effects
Diabetes		Weight loss/gain, cracking body parts	
Diabetes	From what we eat (sweet things)	Frequent urination, over sweating, wounds that never heal, getting ill when one does not eat on time,	Western, private and state, local treatment. These advertise over the radio how they cure diabetes, hypertension and heart failure, spiritual, this covers shrines, churches, combined with western medicine
Diabetes	Legs paralysis, less sugar in take		
Diabetes		Drinking a lot of water, self injection, less sugar in take,	
diabetes		Frequent urination	western
Diabetes (not common among the young ones)			
Diabetes (related to pressure)	Taking sweet food		
Diabetes starts after 40 years	Genetic		
Epilepsy	God made, genetic	Seizures, close to fire	Tradition
Epilepsy	Genetic	Fits, seizures	Herbal western does not treat it
Epilepsy	Genetic	Seizures, energy loss, confusion, mental confusion	Western but herbs heal it
Epilepsy	Witch craft	Seizures, mentally confused	
Epilepsy	Hereditary,	Seizures, convulsions, froth,	Western when they get attacks, nothing, spiritual.
Epilepsy	Brain damage	Seizures, mental confusion, make noise before seizures,	Western, herbalist, prayers but God helps those who help themselves.
Epilepsy		Seizures,	

Epilepsy		Seizures, fats	
Epilepsy	Yaabwe, fever, witchcraft	Seizures and convulsions	Herbs cure it (spoon in the mouth and soda)
Epilepsy	Genetic, contact with saliva from an epileptic victim		Herbs before falling in fire
epilepsy	Witchcraft, heredity,	Seizures, mental confusion,	Western and traditional. Herbs cured it.
Epilepsy		Dizziness, seizures and unconsciousness	Local and western
Epilepsy	Don't know	Seizures, space staring	
Epilepsy	Genetic, witchcraft	Seizures, walking, aimless, tiredness, cough	Spiritual (sheiks, pastors), western medication
Epilepsy		Seizures, froths	
Epilepsy		Seizures	Traditional
Epilepsy	Uncontrolled alcohol consumption, scars and bruises,	Seizures, mental confusion, convulsions, body weakness, froths,	Western but does not cure it. Parents have no way to take them
Epilepsy	Heredity, spirits being unhappy,	Seizures, frothing,	
Epilepsy	Brain failure to work properly,	Seizures,	
epilepsy	Hereditary,	Seizures, mental confusion	Western,
Heart failure	Genetic, worries		
Heart failure	Don't know		Western
Heart failure	Don't know		
Heart failure (related to pressure because all affect the heart.)		Struggles to breath	
HIV	promiscuity	Skin rash, herpes-zoster	Western medicine, churches for those still in denial
HIV	Unprotected sex	Weight loss, sores, loss of appetite, fever, vomiting, energy and blood loss, stroke, palpitations, anaemia	Cost of and out of stocks, western drugs lead people to use herbs
HIV	Unprotected sex	Weight loss, sores, fever, skin, rash, hair loss/texture	Mostly western because you swallow once

HIV		Diarrhoea	
HIV	Un protected sex with an infected person, sharing sharp instruments,	Cough, skin spots, general body weakness, coldness and fever	Western, spiritual but affects treatment adherence
HIV	Sexual unprotected, sharing sharp instruments with infected individuals, blood transfusion unsafe	Hair loss, herpes-zoster, skin rash, red lips	
HIV	Sexual unprotected, accidents	Weight loss, hair loss, red lips, fever, skin rash	Western
HIV	Sexual unprotected, blood transfusion, sharing sharp instruments		Western (doctors shout at patients) and local
HIV	Unprotected sex, alcohol (impaired decision making), panadol and aspirin deter detection of HIV when tested	Western, prayers and local	
HIV	Sexual unprotected, accidents, sharing sharp objects unsterilized	Herpes-zoster, red lips, weight loss, running stomach, appetite loss	
HIV		Boils, strong cough, darkening and falling out nails, fever, red lips, weight loss, rash	western
HIV/AIDS			Western followed by prayers
HIV/AIDS	Unprotected sex with an infected person, witchcraft, accidents	Red lips, skin rashes, fever, cancer	Western, spiritual (combine church and seers)
HIV/AIDS	Carelessness, unfaithfulness, unprotected sex with an infected person, professional exposure e.g. midwives	Red lips, skin rashes, hair loss, headache,	Western, traditional,
HIV/AIDS		Sever cough, flue, daily taking of drugs, red lips, skin rash, herpes-zoster	western
HIV/AIDS	Sexual intercourse (unprotected)		Western

HIV/AIDS	Unprotected sex, accidents, blood transfusion, sharing sharp instruments	Rashes, weight loss, being ill all the time, hair loss,	Western, local and spiritual. Born again claim to cure HIV/AIDS
HIV/AIDS	Unprotected sex win an infected person, accidents,	Slim, weak,	
HIV/AIDS	Witchcraft, unprotected sex with an infected person, unfaithfulness		Western,
HIV/AIDS	Girls fear pregnancy less than HIV, TB linked to HIV		
Hypertension/heart diseases	Being worried about debts, sacked from a job, frightened, extremely happiness,	High palpitations, collapse,	Western and local. Herbalists claim to cure pressure
Hypertension/heart failure	Worrying,	Over pumping of the heart, getting tired quickly, increased breathing rate, easily scared,	Western, traditional
Hypertension/heart failure	Being worried, over thinking,		western
Hypertension/heart failure	Worries, raw salt intake, unbalanced diet. Fat blocking blood flow,	General body weakness, headache, difficulty in breathing,	Western and local, (before diagnosis used to do self medication)
Hypertension/heart failure	Obesity, obstructed blood flow,	high pulse rate, don’t want to be scared,	
Hypertension/lung infection	Cigarette smoking		
Pressure	Black out, being frightened		Local (onions& soda) western
Pressure	Poverty-wealth, stress		
Pressure	Stress, unbalanced diet/ without greens	Weight gain, collapse, hard breathing	Local because you can share with another suffering from a different disease
Pressure	Worries fees, food loans/debts, narrow veins	Blurred vision, sweating, palpitations, general body weakness	Western (drugs are available but health workers come late)
Pressure	Lack of physical exercise, old age		Western and local

Pressure	Old age	Increased breathing rate, dizziness (delayed to seek treatment because much work, wanted a 3rd diagnosis to confirm)	No treatment because no pain as yet
Pressure		Tiredness, headache, feeling frightened, sweating	Western prayers (Pastor advices to go 4 testing) herbs
Pressure		Swollen legs	Stop taking salt and sugar, western, herbs, prayers
Pressure (COPD/lung illness, heart disease)	Worries, drug abuse, avoid frightening, collapse	Over sweating, swollen legs/arms, heat shortly after covering self, heavy breathing, being terrified, blurred vision, paralysis	Western medication, private clinics over charge
Pressure/ heart disease		Feeling scared, sickly, weight gain, palpitations	
Pressure/heart disease		Over sweating, unconsciousness, collapse, headache, leg swelling	Herbal (garlic vanilla seeds, and bitter…) western
Pressure/heart failure	Stress, worrying,	General body weakness, bad vision,	Western and traditional. Grade be always advised to take him the following day.
Pressure/heart failure	Worrying too much, eating raw salt, use of family planning,	Over sweating, feel hot most of the time, high palpitations	Western,
Pressure/heart failure	Thinking hard	Stroke, unconsciousness, heart pains, feeling frightened, breathing fast	Western
TB/ Asthma/lung infection	Exposing babies to cold weather, hereditary, marijuana/cigarette smoking	cough	Western, traditional medicines,
TB/Asthma	Smoking which causes holes in the lungs	Nose congestion, difficulty in breathing, flue during cold weather,	Western,
TB/Asthma/lung infection	Cough when mouths are not covered, cold weather, smoking,	Cough, chest pain, wearing many clothing, breathing difficulties,	Take long to heal, western
